# *Aeromonas hydrophila* RIT668 and *Citrobacter portucalensis* RIT669—Potential Zoonotic Pathogens Isolated from Spotted Turtles

**DOI:** 10.3390/microorganisms8111805

**Published:** 2020-11-17

**Authors:** Seema G. Thomas, Milky Abajorga, Maryah A. Glover, Peter C. Wengert, Anutthaman Parthasarathy, Michael A. Savka, Crista B. Wadsworth, Paul A. Shipman, André O. Hudson

**Affiliations:** Thomas H. Gosnell School of Life Sciences, Rochester Institute of Technology, Rochester, NY 14623, USA; sgtsbi@rit.edu (S.G.T.); milky.abajorga@umassmed.edu (M.A.); mag3461@g.rit.edu (M.A.G.); pcw4943@g.rit.edu (P.C.W.); axpsbi@rit.edu (A.P.); massbi@rit.edu (M.A.S.); cbwsbi@rit.edu (C.B.W.); passbi@rit.edu (P.A.S.)

**Keywords:** *Aeromonas hydrophila*, *Citrobacter portucalensis*, antibiotics, antibiotics resistance, quorum sensing

## Abstract

Antimicrobial resistance (AMR) is one of the biggest challenges of the 21st century, and biofilm formation enables bacteria to resist antibiotic at much higher concentrations than planktonic cells. Earlier, we showed that the Gram-negative *Aeromonas hydrophila* RIT668 and *Citrobacter portucalensis* RIT669 (closely related to *C. freundii* NBRC 12681) from infected spotted turtles (*Clemmys guttata*), formed biofilms and upregulated toxin expression on plastic surfaces, and were predicted to possess multiple antibiotic resistance genes. Here, we show that they each resist several antibiotics in the planktonic phase, but were susceptible to neomycin, and high concentrations of tetracycline and cotrimoxazole. The susceptibility of their biofilms to neomycin and cotrimoxazole was tested using the Calgary device. For *A. hydrophila*, the minimum inhibitory concentration (MIC) = 500–1000, and the minimum biofilm eradication concentration (MBEC) > 1000 μg/mL, using cotrimoxazole, and MIC = 32.3–62.5, and MBEC > 1000 μg/mL, using neomycin. For *C. freundii* MIC = 7.8–15.6, and, MBEC > 1000 μg/mL, using cotrimoxazole, and MIC = 7.8, and MBEC > 1000 μg/mL, using neomycin. Both *A. hydrophila* and *C. portucalensis* activated an acyl homoserine lactone (AHL) dependent biosensor, suggesting that quorum sensing could mediate biofilm formation. Their multidrug resistance in the planktonic form, and weak biofilm eradication even with neomycin and cotrimoxazole, indicate that *A. hydrophila* and *C. portucalensis* are potential zoonotic pathogens, with risks for patients living with implants.

## 1. Introduction

Antibiotic resistance is an emerging crisis in bacterial diseases, as the frequency of resistance in clinical as well as environmental settings increases, and the development of new antibiotics slows down [1,2,3]. *Aeromonas* and *Citrobacter* are two widely distributed Gram-negative genera containing several pathogenic species of clinical concern. Of these, *Aeromonas hydrophila* and *Citrobacter freundii* strains are considered emerging pathogens [4,5], which can both form biofilms [6,7,8,9]. *A. hydrophila* and *C. freundii* infect both freshwater and marine turtles, and antibiotic resistance among the turtle strains has been reported [10,11,12]. Turtles have been regarded as “sentinel species” for assessing ecosystem health [13,14] and the illegal trade in reptiles at “wet markets” could pose a significant risk of zoonotic transmission [15,16]. In an earlier study, we reported the biofilm formation and Shiga-toxin expression by *A. hydrophila* and *C. freundii* strains isolated from infected spotted turtles (*Clemmys guttata*) [17]. In this study, we have reclassified our *C. freundii* strain whose classification was based on the 16S-rDNA gene phylogeny, as a *C. portucalensis* strain on the basis of whole genome comparison. Bacteria associated with reptiles could infect warm-blooded mammals, since reptile and clinical strains of *Aeromonas* spp. were identical in some cases [18].

In *Aeromonas*, acquired resistance increases the level of antibiotic resistance in both environmental and clinical strains [19]. The transfer of extended spectrum β-lactamase genes from enterobacteria to *Aeromonas* via plasmids is known [20]. Resistance to β-lactams, quinolones, macrolides, tetracyclines, sulfonamides, and chloramphenicol may be conferred by transposons, integrons, and plasmids [21,22]. Although *Aeromonas* spp. can cause various types of pathologies, they are emerging as an enteric pathogen of public health concern [4].

*Citrobacter portucalensis* was first reported from aquatic ecosystems [23], but multidrug resistant strains have been reported from poultry [24] and green leafy vegetables [25]. The closely related *Citrobacter freundii* is increasingly a nosocomial and environmental pathogen which causes pneumonia, diarrhea, urinary tract, bloodstream, and invasive brain infections [5,26,27,28,29,30,31,32,33,34]. Foodborne, hospital and pediatric outbreaks, neonatal infections in preterm infants and hemolysis along with diarrhea, were also reportedly caused by *C. freundii* strains [26,28,35,36,37,38]. Antibiotic resistance in *C. freundii* strains is increasing, and extended β-lactamase and plasmid-mediated quinolone resistance have been reported [32,39,40,41,42,43]. *C. freundii* strains have been isolated from mixed biofilms alongside strains such as *A. hydrophila*, *Enterobacter soli*, and *Stenotrophomonas maltophilia* [6]. The presence of *C. freundii* worsens existing *Pseudomonas aeruginosa* infections in murine models and also likely in patients with co-infection [7].

While resistance is typically studied in planktonic cells, multiple research groups have shown that biofilms can exhibit a greater than hundred-fold increase in resistance to antibiotics compared to the same bacteria in a planktonic state [44,45,46]. This could be the reason for the frequent lack of correlation between in vitro antimicrobial studies and therapeutic results [12]. Biofilm-associated cells differ from planktonic cells due to the generation of an extracellular polymeric substance (EPS) matrix, reduction in growth rate, and the altered regulation of genes [47]. These infections routinely colonize in-dwelling medical devices (IMDs) [48,49] such as abdominal [50] and coronary stents [51], and are more persistent and complicated to treat than infections caused by planktonic cells [46,52]. This is thought to be due to the physiological alteration of the bacteria upon attachment to a surface, as well as to the cell specialization that occurs within biofilms [53,54,55]. The enhanced virulence of biofilms, their impacts on IMDs, and their relevance for therapeutic success all point to the importance of assessing antibiotic resistance in the biofilm phase. Furthermore, the density-dependent mechanisms of acyl-homoserine lactone (AHL) regulated expression in the process called quorum sensing (QS), which is known to be associated directly with clinical antibiotics carbapenem and mupirocin and other natural products, bactobolin and enacyloxin, that possess antibiotic activities [56,57,58,59].

Both *A. hydrophila* and *C. freundii* have been shown to be susceptible to neomycin and cotrimoxazole in the planktonic phase [60,61,62,63,64]. We have previously predicted using whole genome sequencing that *A. hydrophila* RIT668 contains resistance genes for six classes of antibiotics, while *C. freundii* RIT669 contains resistance genes for 19 classes of antibiotics [17]. In the same report, we also showed the growth of biofilms of *A. hydrophila* RIT668 and *C. freundii* (now *C. portucalensis*) RIT669, and the upregulated expression of a Shiga-like toxin during adhesion on the polymers, polyethylene (PE) and polypropylene (PP) [17]. However, experimental verification of the antibiotic resistance in the planktonic and biofilm forms was missing. Therefore, we investigated the antibiotic resistance patterns of *A. hydrophila* RIT668 and *C. portucalensis* RIT669, and report resistance against gentamicin, tetracycline, doxycycline, kanamycin, streptomycin, tobramycin, penicillin, erythromycin, and novobiocin, in the planktonic phase. Both bacteria are susceptible to cotrimoxazole (sulfamethoxazole-trimethoprim dosed 5:1) and neomycin in the planktonic phase.

We measured the minimum inhibitory concentration (MIC), and the minimum biofilm eradication concentration (MBEC) values for the antibiotics, cotrimoxazole and neomycin, for these two bacteria. The MBEC can be hundreds or thousands of times higher than the MIC for the same antimicrobial and the same target microorganism [44,46,65,66,67,68,69]. The MBEC assay in the 96-well format is a frequently used high throughput method to test for biofilm eradication, which is based on the Calgary biofilm device [44], which allows convenient testing over a wide concentration range (low microgram to low milligram in our panel).

## 2. Materials and Methods

### 2.1. Bacterial Isolates and Background

Microbial samples were isolated from 12 rescued adult spotted turtles (*Clemmys guttata*). The spotted turtle is a small and semi-aquatic species found in North American and is often illegally captured for the pet trade [70,71]. The animals in this study were rescued by the United States Fish and Wildlife Service during a raid on an illegal reptile trading operation (chain of custody ID number-ST#032797). The animals in this study were infected, lethargic and showed reduced/stopped food intake for a week. Eye inflammation was observed, and a slimy secretion surrounded the eyes, nostrils and feet of the spotted turtles. All the animals were infected by *A. hydrophila* and *C. freundii*. Starting from a 50% mortality rate at the beginning of the study, all animals had succumbed by the end of the study (mortality rate 100%). The bacteria in this study were swabbed from the eyes, nostrils, and feet of infected hatchling and adult rescued spotted turtles (*Clemmys guttata*) under captivity. The samples were swabbed onto LB, Blood agar plates (5% sheep blood), and MacConkey plates as to enrich the growth of multiple types of fastidious strains. The plates were incubated overnight at 37 °C and re-streaked on fresh plates to obtain pure cultures. For calculating the doubling times in liquid media, the cells were grown in tryptic soy broth (TSB) at 37 °C and the absorbance at 600 nm recorded.

### 2.2. Characterization and Identification: Biochemical Assay and 16S rDNA Amplification

Primary identification via Grams staining, oxidase and catalase tests were performed followed by the five groups of biochemical assays for microbial identification: Group 1—glucose, gas, lysine; Group 2—ornithine, H_2_S, indole; Group 3—adonitol, lactose, arabinose; Group 4—sorbitol, voges prousker, dulcitol; and Group 5—phenylalanine (PA), urea, citrate. The bacteria, two strains were identified via 16S amplification and used in the current study. For the 16S rDNA amplification, the microbial DNA was isolated using the ‘UltraClean Microbial DNA Isolation Kit’ from MO BIO Laboratories Inc. Colony PCR was performed using the primers 341F = 5′ CCTACGGGNGGCWGCAG 3′ and 805R: 5′ GACTACHVGGGTATCTAATCC 3′ (where N = any nucleotide, W = A or T; H = A, C, or T; V = A, C, or G), for the V3–V4 region using the GoTaq Green Mix containing the GreenTaq polymerase from Genescript Inc. (Piscataway, NJ, USA). The PCR conditions were, denaturation at 95 °C for 5 min, followed by 30 cycles at 94 °C for 1 min, 50 °C for 1 min (annealing), 72 °C for 1 min, and a final extension at 72 °C for 10 min. Amplified products were purified using an EZ-10 Spin Column PCR Products Purification Kit (Bio Basic Canada Inc., Markham, ON, Canada), and sequenced with 341F as the sequencing primer at the Genewiz sequencing facility, NJ, USA. The sequences generated were compared using the Basic Local Alignment Search Tool (blastN) [72] to identify the genera using the default parameters.

### 2.3. Supplemental Bioinformatics Methods

The assembled genomes of RIT668 and RIT669 were uploaded to the Type Strain Genome Server (TYGS) on 2020.11.04. TYGS generates whole genome-based taxonomies and digital DNA–DNA hybridization values for submitted genome assemblies [73]. Briefly, a list of closely related strains was determined based on the 16S sequence extracted from the query genome via RNAmmer [74]. GBDP distances were calculated for genome pairs with close 16S matches [75]. The shortest GBDP distances were used to determine the 10 closest type strains to the query. Mash (a whole-genome clustering method) then determined closest strains to the query strain from a type-strain database [76]. Based on the pairwise digital DNA–DNA hybridization values, TYGS generated a genome-based phylogenetic tree containing the query genome and closely related prokaryotic genomes using FASTME 2.1.4 [77]. Branch lengths were calculated from 100 bootstrap replicates.

To further establish the taxonomic assignment of RIT669, the FASTA assembly of RIT669 was uploaded to JSpecies, a free, web-based service to calculate average nucleotide identity between RIT669 and type strains of *Citrobacter portucalensis* and *Citrobacter freundii* [78]. ANI calculations were made between RIT669, *C. portucalensis* and *C. freundii* using both BLAST+ and MUMmer methods [79].

### 2.4. Antimicrobial Susceptibility/Multidrug Resistance Based on Disc Diffusion Assays

#### 2.4.1. Disc Diffusion with Pre-Loaded Discs

Antibiotic susceptibility was quantified using pre-loaded discs containing different antibiotics as follows: gentamicin (10 μg), tetracycline (30 μg), doxycycline (30 μg), kanamycin (30 μg), streptomycin (10 μg), tobramycin (10 μg), neomycin (30 μg), novobiocin (30 μg), erythromycin (15 μg), and sulfamethoxazole (sulfamethoxazole 23.75 μg + trimethoprim 1.25 μg). In brief, Mueller–Hinton plates were inoculated with 100 μL of overnight cultures of *A. hydrophila* and *C. portucalensis* at 37 °C. After 24 h, pre-loaded antibiotic discs from (BD Biosciences, East Rutherford, NJ) were placed on each plate and incubated overnight at 37 °C. After the incubation, the zones of inhibition were evaluated based on the Clinical and Laboratory Standards Institute (CLSI) standards for Enterobacteriaceae [80]. In the absence of CLSI data, the European Committee on Antimicrobial Susceptibility Testing (EUCAST) breakpoint recommendations were used [81].

#### 2.4.2. Disc Diffusion with Stock Antibiotic Concentrations

The high concentration tests were prepared as follows: 40 mL tryptic soy agar (TSA) was poured and inoculated with 0.4 mL of overnight culture at OD_595_ of 0.5 of *A. hydrophila* or *C. portucalensis*. After drying and cooling, 6 mm sterile blank paper discs (BD Biosciences, San Jose, CA, USA) were placed on the agar plates. Concentrations for each antibiotic are indicated in brackets: tetracycline (30 mg/mL), rifamycin (30 mg/mL), cefaclor (cephalosporin; 50 mg/mL), fusidic acid (12.5 mg/mL), kanamycin (50 mg/mL), clindamycin (50 mg/mL), neomycin (30 mg/mL), and cotrimaxazole (sulfamethoxazole:trimethoprim = 1:5; 30 mg/mL). Each disc was spotted with 20 μL of an antibiotic stock solution from those listed above. A control disc with sterile water was used.

### 2.5. Biofilm Formation and Biofilm Growth Check

Cultures of both *A. hydrophila* and *C. portucalensis* at a cell density of 10^6^ CFU/mL were added to two individual trough bases and incubated at 37 °C on a rocking platform. The established biofilms on peg lids were transferred to a series of 96 well plate for testing the MBEC, and MIC, alongside sterile control wells. A serial dilution of 10^−1^ to 10^−7^ was prepared by transferring 20 μL to each of the eight rows of the 96-well plate. 20 μL was removed from each well and spot plated onto the TSA plates. The 96-well plate was then incubated on a rotary shaker at 37 °C for 16 h. Biofilm growth check was performed and immediately following incubation, specified pegs were removed from the lids using flame-sterilized pliers and each were placed in a new 96-well plate with recovery media. The plate was sonicated for 30 min to recover the biomass. The cell density was confirmed by serial dilution and spot plating.

### 2.6. MIC and MBEC Assay Device

**Figure 1:**
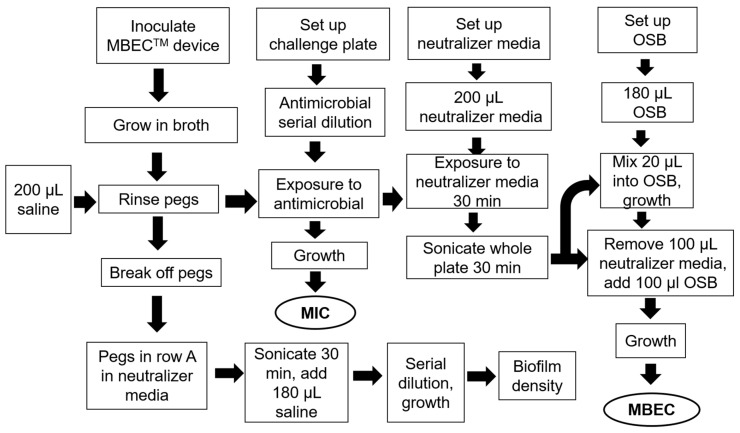
Schematic representation of the high throughput in vitro antimicrobial testing model to evaluate efficacy of cotrimoxazole (sulfmethoxazole and trimethoprim) and neomycin, estimated via the MIC and MBEC values against *A. hydrophila* and *C. portucalensis*. OSB refers to organism specific broth; in this work trypticase soy broth (TSB) was used.

To asses both the MICs of planktonic cells and the efficacy of antimicrobials against biofilm we used the MBEC assay device (Innovotech Inc, Edmonton, AB, Canada). In brief, second sub-cultures of *A. hydrophila* and *C. portucalensis* were grown in TSB media were adjusted to a cell density of 10^6^ CFU/mL. The MBEC, MBC, and MIC were analyzed following the standard protocol as per Innovotech’s MBEC assay (Innovotech Inc, Edmonton, AB, Canada), https://www.innovotech.ca/wp-content/uploads/2019/02/MBEC-Procedural-Manual-v2.0.pdf. The assay workflow is presented in Figure 1. The antibiotic concentrations used in each of the eight rows of the 96-well plate are presented in the legends to Figure 1. The challenge plate was prepared by diluting a 1 mg/mL stock solution of neomycin or cotrimoxazole in each row. TSB, sterile neutralizer and water were added to three specific wells designated the sterile, neutralizer, and neutralizer effective control. It was freshly prepared and used within 30 min. The rinse plate was prepared by adding 180 µL of TSB to a new 96-well plate. The peg lid was rinsed by setting the lid into rinse plate for 10 s. The peg lid was transferred to the challenge plate and incubated as per the protocol. The recovery plate was prepared by adding 200 μL of neutralizer media to a new plate and after the appropriate time the peg lid was transferred to the recovery plate. The device was again sonicated to remove the attached biofilm. 100 μL of sterile media was added to each well of recovery plate and incubated at 37 °C for 24 h to determine the MBEC via a micro-titer plate reader at 650 nm. The MBEC value is the minimum concentration of antibiotic that inhibits growth of biofilm as indicated by the control wells with no turbidity. The MBEC protocol used was modified minimally from the manufacturer’s manual neomycin and cotrimoxazole (trimethoprim/sulfamethoxazole) serially diluted with the highest concentration at 1 mg/mL each, were used as antibiotics, with each of the eight rows of the 96-well plate, from A through H, being 1000, 500, 250, 125, 62.5, 31.3, 15.6, and 7.8 µg/mL, respectively.

The MBEC and MIC were analyzed following the standard protocol as per Innovotech’s MBEC assay. The MBEC assay workflow is presented in Figure 1.

### 2.7. Scanning Electron Microscopy (SEM)

The *A. hydrophila* and *C. portucalensis* biofilm-covered pegs treated with cotrimoxazole and neomycin were rinsed with phosphate buffered saline (PBS) and 2% glutaraldehyde. The samples were further processed according to an open source protocol [82]. After sputter-coating for two min with gold-palladium, the SEM was performed at a voltage of 5 kV using a Mira3 Tescan field emission SEM at the Rochester Institute of Technology (RIT) Nanoimaging Lab.

### 2.8. Identification of Quorum Sensing Signals of the Acyl-Homoserine Lactone (AHL) Class

Complementary formats [83,84] were used to identify then to separate and identify quorum sensing signals of the acyl-homoserine lactone (AHL) class. Plate T-streak and diffusion-disc bioassays are standard protocols in our laboratory and performed as previously described [85,86]. Reverse phase (RP) thin layer chromatogram was performed with silica gel 60 RP-18 plates (20 × 20 cm, EMD Chemical Inc., Gibbstown, NJ, USA). For RP TLC every 2.8 cm on the line, a mark was made to identify the center of each column. Each samples was loaded with 2–3 µL volumes intervals to reach the final sample size. Conditions of TLC development (60% methanol:water), and the preparation and use of whole cell AHL-dependent bacterial biosensors and visualization of AHL signals were as described previously [84,85]. After drying, the position and number of AHLs were detected with a thin overlay of the TraR-dependent *Agrobacterium* biosensor strain A136 [87]. The biosensor-overlaid plate was left in a sterile hood to solidify and dry for 30 min, then covered and placed in a 28 °C incubator overnight [84,85,86].

### 2.9. Bioinformatics Analysis of AHL-Related Genes

The genomes of strains *A. hydrophila* RIT 668 and *C. portucalensis* RIT669 were scanned for sequences homologous to established quorum sensing genes *luxI* and *luxR*. This was performed by first mining each genome for proteins using Prodigal [88], and subsequently using HMMER to scan the predicted protein database using PFAM domains PF03472 and PF0076 as bait for LuxR and LuxI, respectively [89]. The HMM hits were further scrutinized using InterProScan7 [90]. Hits showing domains IPR016032, IPR005143, and IPR000792 were considered to be valid LuxR homologs, while hits showing domains IPR001690 and IPR018311 were considered to be valid LuxR homologs, as described in Gan et al. [91].

### 2.10. Computational Methods

The doubling times in TSB was calculated using the online tool https://doubling-time.com/compute.php [92], and was reported as an average of the doubling time values at different time intervals after inoculation, such as 35, 85, 115, 130, 145, 165, and 225 min, during the exponential phase. The zones of inhibition were recorded and categorized as—resistant (R), intermediate (I) and susceptible (S). Statistical analysis of the MIC, MBC, and MBEC results were performed using ANOVA with α = 0.05 and Fisher’s (95% confidence intervals).

## 3. Results

### 3.1. A. hydrophila

#### 3.1.1. Growth

Doubling time analysis of our *A. hydrophila* strain when grown in tryptic soy broth at 37 °C showed an average generation time of 94 min. An earlier work on the growth at 35 °C of *A. hydrophila* strains in Brain Heart Infusion broth buffered with 3-[N-morpholino] propane sulfonic acid (MOPS) and adjusted to pH 7.0, reported generation times of 19.8–23.4 min [93].

#### 3.1.2. Taxonomy

The assembled genomes of RIT668 was placed within taxonomic contexts through a whole-genome approach via the Type Strain Genome Server (TYGS). Strain RIT668 grouped most closely with a type strain of *Aeromonas hydrophila* ATCC 7966 with a bootstrap support value of 96 on the Genome Blast Distance Phylogeny tree (GBDP), as shown in Figure S1. The phylogenetic tree was created using GBDP distances between RIT668 and 15 closely related genomes in the TYGS database, and had an average branch support of 99.0% with a δ statistic of 0.166, a measure of tree-likeness. The assembly of RIT 668 had a dDDH d_4_ percentage of 74.9% compared to the genome of *Aeromonas hydrophila* ATCC 7966 and 72.2% compared to the genome of *Aeromonas hydrophila* subsp. ranae CIP 107985. The d_4_ value was chosen to avoid issues caused by an incompletely sequenced genome. The accepted dDDH threshold for two genomes coming from the same species is 70%.

#### 3.1.3. Antimicrobial susceptibility screening with preloaded antibiotic discs

The disc diffusion assay was applied as a preliminary analysis to determine the effect of 11 different antibiotics (Table 1) on the susceptibility of *A. hydrophila*, under standard conditions, using fixed concentration paper antibiotic discs along with a control. *A. hydrophila* was resistant to gentamicin (10), tetracycline (30), doxycycline (30), kanamycin (30), streptomycin (10), tobramycin (10), novobiocin (30), and erythromycin (15 µg), respectively. *A. hydrophila* was susceptible only to neomycin and sulfamethaxazole-trimethoprim.

#### 3.1.4. Antimicrobial Susceptibility Screening Using the Stock Concentration Method

Disc assays using stock concentrations of selected antibiotics did not have an inhibitory effect in most cases on *A. hydrophila* grown on TSA. Only tetracycline, neomycin and cotrimoxazole had significant inhibition zones (Table 2). The drugs tested belongs to aminoglycoside, tetracycline, aminocoumarin, macrolide, beta-lactam, ansamycin, cephalosporin, fusidane, lincomycin, and sulfonamide classes, out of which our isolates are resistant to the aminoglycoside (except neomycin), fusidane, lincomycin, ansamycin and cephalosporin classes (Table 3). *A. hydrophila* was susceptible to neomycin, cotrimoxazole, and tetracycline, which target the 30S ribosomal subunit preventing protein synthesis, the enzyme dihydropteroate synthase (DHPS), and the binding of aminoacyl tRNA to the RNA-ribosome complex, respectively.

#### 3.1.5. MIC and MBEC

**Figure 2:**
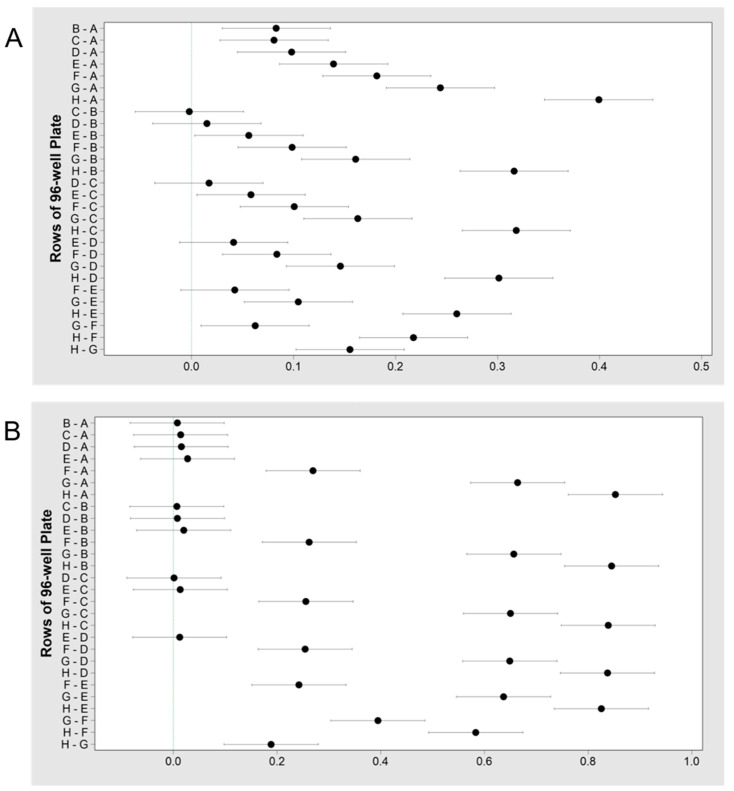
Fisher individual 95% confidence intervals for the MIC values from the challenge plate of *A. hydrophila* containing (**A**) cotrimoxazole; the MIC is determined to be between 1000 and 500 μg/mL (rows A and B) due to lack of growth and a significant difference in the absorbance between rows A and B, but not rows B and C, (**B**) neomycin; the MIC is determined to be between 62.5 and 32.3 μg/mL (rows E and F) due to lack of growth and a significant difference in the absorbance between rows E and F.

MIC: The MIC of *A. hydrophila* when challenged with the antibiotic cotrimoxazole was found to be between 500 and 1000 μg/mL (Table 4); whereas the MIC with Neomycin for this same bacterium was found to be between 32.3 and 62.5 μg/mL (*P* < 0.05, Table 4). An exact numerical value could not be determined due to the nature of the protocol as the concentrations decreased in each row by a factor of two. Values shown in the MIC ranges were determined as the lowest concentration of the antibiotic where microbial growth was inhibited and the Fisher confidence intervals are shown for cotrimoxazole (Figure 2A) and neomycin (Figure 2B).

MBEC: A trend indicating increased biofilm eradication at higher antibiotic concentrations was observed. Cotrimoxazole and neomycin in the concentration range of 7.8 to 1000 μg/mL, were able to eradicate biofilms of both *A. hydrophila* (*P* > 0.05). Between 125 and 250 μg/mL of cotrimoxazole, growth drastically decreased, but biofilms were not eradicated (Figure S2A). Similar results were observed for neomycin (Figure S2B). This could be due to the highest concentration being as low as 1 mg/mL. Overall, the trend showed neomycin treatment with higher concentrations were generally more effective than cotrimoxazole.

#### 3.1.6. Scanning Electron Microscopy (SEM) Analysis of MBEC Samples

**Figure 3:**
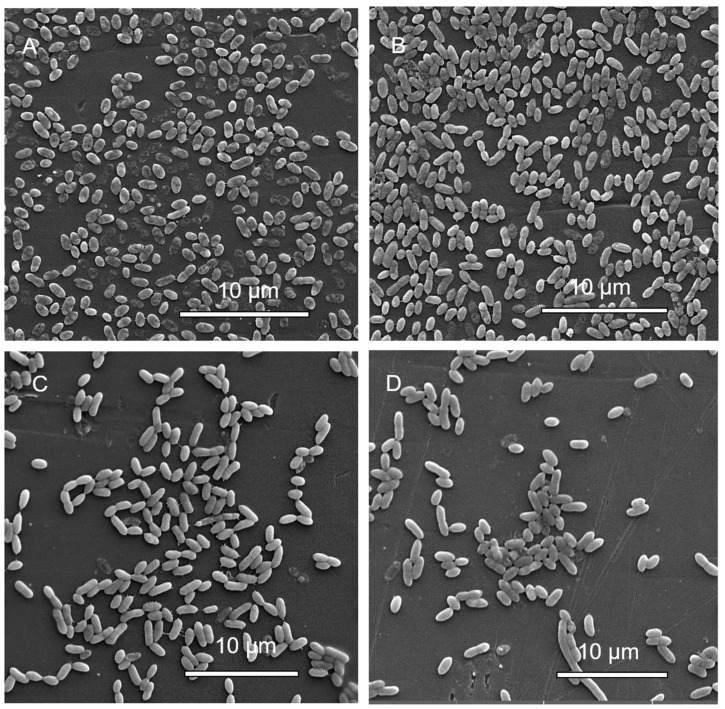
Scanning electron micrographs of *A. hydrophila* biofilm inoculation pegs at (**A**) the lowest concentration of cotrimaxazole (7.8 μg/mL; magnified 7070×), (**B**) the highest concentration of cotrimaxazole (1000 μg/mL; magnified 7120×), (**C**) the lowest concentration of neomycin (7.8 μg/mL; magnified 6540×), and (**D**) the highest concentrations of neomycin (1000 μg/mL; magnified 6470×).

SEM imaging of *A. hydrophila* revealed fewer cells in samples containing highest concentrations of antibiotics, and higher number of cells in samples exposed to lowest concentrations of antibiotics (Figure 3). *A. hydrophila* treated with cotrimoxazole resulted in biofilms showing multiple layers of cells at the lowest antibiotic concentration (Figure 3A) and a single layer at the highest concentration (Figure 3B), while neomycin-treated samples showed flat biofilms in the presence of both the highest and lowest antibiotic concentrations (Figure 3C,D).

#### 3.1.7. Detection of Quorum Sensing

**Figure 4:**
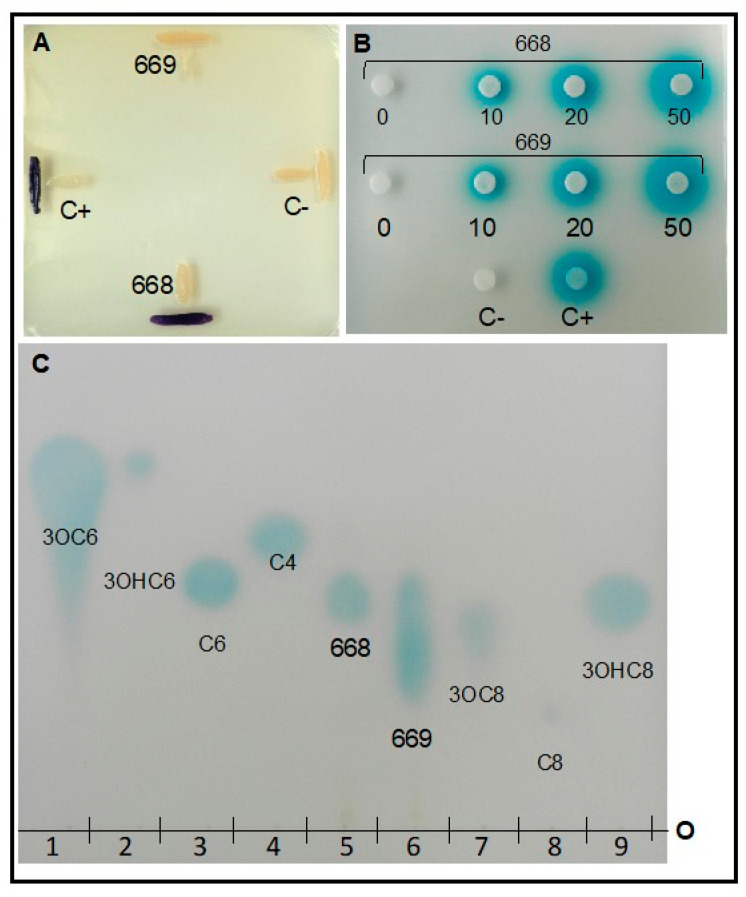
Quorum sensing signals of the acyl-homoserine lactone (AHL) class are produced by *A. hydrophila* RIT668 and *C. portucalensis* RIT669. Three formats using two complementary AHL-dependent whole cell biosensor strains were used, *Chromobacterium violaceum* CV026 (panel **A**) and *Agrobacterium tumefaciens* NTL4 (pZLR4) (panels (**B**,**C**)) [70]. T-streak bioassay using biosensor CV026 (A) C+ strain, DH5a (pT140A) and C− strain, CV026. Disc-diffusion bioassay using increasing volumes of ethyl acetate extract from early stationary stage cultures of *A. hydrophila* (668) and *C. portucalensis* (669), C+ *N*-(3-Oxooctanoyl)-L-homoserine lactone (3OC8), 4 µL 10 nm; C− 30 µL ethyl acetate (**B**); and Thin layer chromatography separation and detection of AHL signals from ethyl acetate extracts of cultures of *A. hydrophila* (668) and *C. portucalensis* (669) (**C**). AHL standards include: lane 1, *N*-(3-Oxohexanoyl)-L-homoserine lactone, (3OC6) (4 µL, 100 nM); lane 2, *N*-(3-Hydroxyhexanoyl)-L-homoserine lactone (3OHC6) (10 µL, 1 mM); lane 3, *N*-Hexanoyl-L-homoserine lactone (C6) (2 µL, 1 mM); lane 4, *N*-Butyryl-L-homoserine lactone (C4) (6 µL, 1 mM); lane 5, RIT668 ethyl acetate extract (10 µL of 200× concentrate); lane 6, RIT669 ethyl acetate extract (10 µL, 200× concentrate), lane 7, *N*-(3-Oxooctanoyl)-L-homoserine lactone (3OC8) (5 µL, 10 nM); lane 8, *N*-Octanoyl-L-homoserine lactone (C8) (10 µL, 100 mM); lane 9, *N*-(3-Hydroxyoctanoyl)-L-homoserine lactone (3OHC8) (4 µL, 1 mM). C+ = positive control; C− = negative control; 668 = *A. hydrophila* RIT668, and 669 = *C. portucalensis* RIT669.

*A. hydrophila* produces AHL-type quorum sensing (QS) signals as assessed by the use of two complementary whole-cell biosensors, CV026 and NTL4 (pZLR4) in T-streak, diffuse-disc and thin layer chromatography formats (Figure 4). Using a narrow- and broad-range AHL-detection biosensor, *A hydrophila* was shown to activate of the reporter genes indicating AHL production. TLC analysis of ethyl acetate extracts from *A. hydrophila* showed the production of a single detectable AHL that co-migrates with *N*-(3-hydroxyoctanoyl)-L-homoserine lactone standard.

### 3.2. C. portucalensis

#### 3.2.1. Growth

The average doubling time at 37 °C in TSB, of our *C. portucalensis* strain was 103 min. This is much slower than the reported doubling time of 50 min for a clinical strain *C. freundii* GN346 at the same temperature [84].

#### 3.2.2. Taxonomy

Based on a GBDP tree, RIT669 was most closely related to *Citrobacter portucalensis* A60, as shown in Figure S3. The GBDP tree containing RIT669 and 13 closely related genomes had an average branch support of 91.7% and a δ statistic of 0.113. The dDDH d_4_ percentage between the assembly of RIT699 and *C. portucalensis* A60 was 80.1%, well about the 70% threshold for conspecifics. Additionally, the dDDH d_4_ value comparing RIT669 and *Citrobacter freundii*, the next most closely related species, was 58.3%.

To further confirm the placement of RIT669 in the species *C. portucalensis*, the assembly of RIT669 was uploaded to JSpecies, an online service for calculation of Average Nucleotide Identity values. The ANIb value comparing RIT669 with *C. portucalensis* strain 4 7 47CFAA was 98.98%, which was above the 95% cutoff for conspecifics. The ANIb value comparing RIT669 with *C. freundii* strain CAV1321 was 94.13%. The ANIm values also supported assignment of RIT669 to the species *C. portucalensis*, with a value of 99.27% between RIT669 and *C. portucalensis* strain 4 7 47CFAA, and a value of 94.67% between RIT669 and *C. freundii*.

#### 3.2.3. Antimicrobial Susceptibility Screening with Preloaded Antibiotic Discs

*C. portucalensis* was resistant to gentamicin (10), tetracycline (30), doxycycline (30), kanamycin (30), streptomycin (10), tobramycin (10), novobiocin (30), and erythromycin (15 µg), respectively. The response to sulfamethoxazole-trimethoprim is characterized as intermediate. However, it is susceptible to neomycin (Table 1).

#### 3.2.4. Antimicrobial Susceptibility Screening Using the Stock Concentration Method

Like *A. hydrophila*, *C. portucalensis* also resisted all the tested antibiotics at the stock concentrations while growing on TSA, except for tetracycline, neomycin, and cotrimoxazole had significant inhibition zones (Table 2). *C. portucalensis* was susceptible to neomycin, cotrimoxazole, and tetracycline, all of which target the 30S ribosomal subunit.

#### 3.2.5. MIC and MBEC

**Figure 5:**
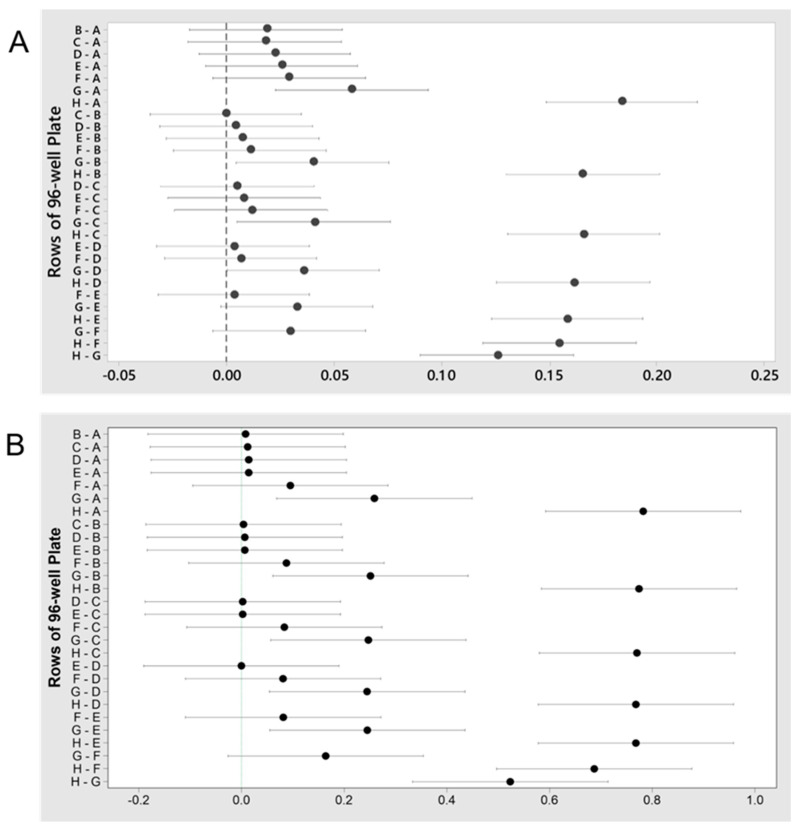
Fisher individual 95% confidence intervals for the MIC values from the challenge plate of *C. portucalensis* containing (**A**) cotrimoxazole; the MIC is determined to be between 15.6 and 7.8 μg/mL (rows G and H) due to effect of antibiotic causing a lack of growth and a significant difference in the absorbance between rows G and H; (**B**) neomycin; the MIC is determined to be between 31.3 and 7.8 μg/mL (rows F and H) due to a lack of growth. More data is required to determine if the MIC is between 31.3 and 15.6 μg/mL (rows F and G) due to near significant differences in the absorbance between rows F and G.

MIC: The MIC for *C. portucalensis* after challenged with cotrimoxazole was determined to be between 7.8 and 15.6 μg/mL (*P* < 0.05) (Figure 5A). For the neomycin test, the concentration was found to be between 7.8 and 31.3 μg/mL (*P* < 0.05) (Figure 5B).

MBEC: Neither of the antibiotics were able to significantly eradicate the biofilms of *C. portucalensis*, at the evaluated concentrations of 7.8–1000 μg/mL (*P* > 0.05) (Figure S4A,B). Higher concentrations were more effective at biofilm elimination; however, eradication was not observed. Overall, both antibiotics were more effective against the biofilms of *C. portucalensis* than those of *A. hydrophila*. The true values of MBEC are probably much higher, since the complete eradication of biofilms was not observed at 1000 µg/mL.

#### 3.2.6. Scanning Electron Microscopy (SEM) Analysis of MBEC Samples

**Figure 6:**
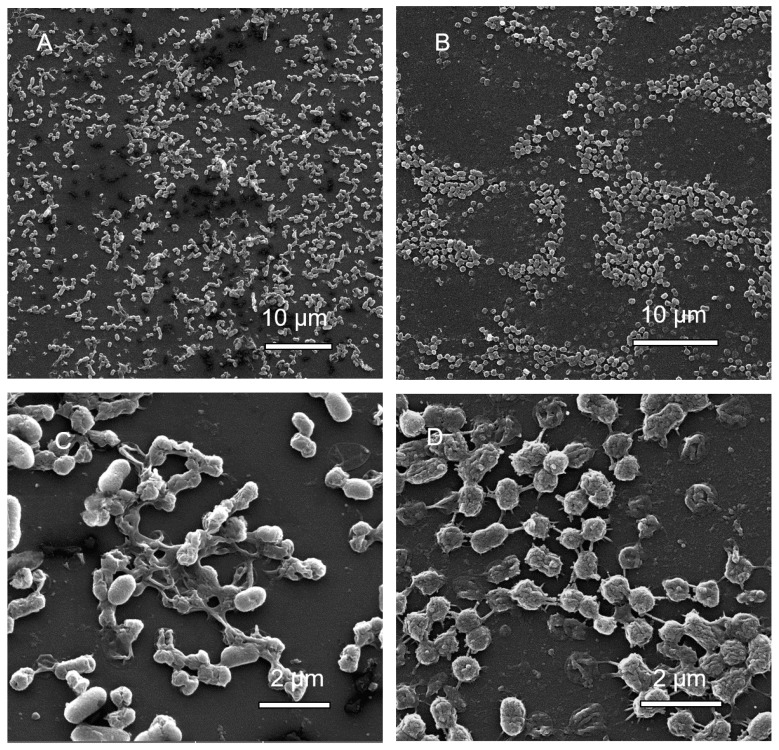
Scanning electron micrographs of *C. portucalensis* biofilm inoculation pegs at (**A**) the lowest (7.8 μg/mL) and (**B**) the highest (1000 μg/mL) concentrations of cotrimoxazole. The images were magnified 3380× and 4430×, respectively. (**C**) (18,700×), and (**D**) (21,300×), show further magnification of cells in (A,B), which received the lowest and highest cotrimoxazole concentrations, respectively.

**Figure 7:**
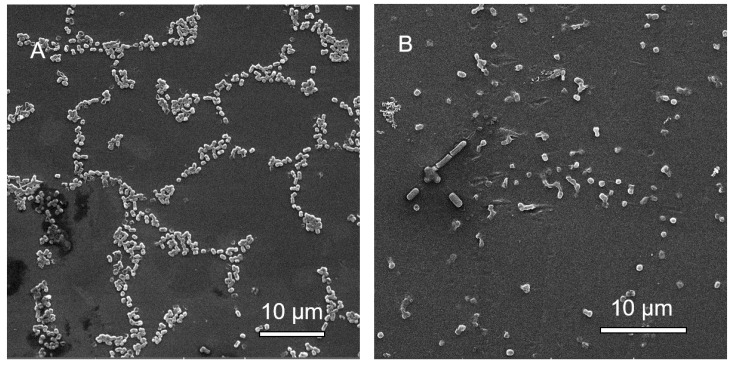
Scanning electron micrographs of *C. portucalensis* biofilm inoculation pegs at (**A**) the lowest (7.8 μg/mL), and (**B**) the highest (1000 μg/mL) concentrations of neomycin. The images are magnified 3600× and 4880×, respectively.

Most samples did not show major morphological changes, except *C. portucalensis* treated with cotrimoxazole. At the lowest concentrations, biofilm formation can be seen to contain cells with projections (Figure 6A). The cells at the highest concentration occur in a flatter arrangement (Figure 6B), but the cells appear linked by fibers under higher magnification (Figure 6C,D). Additionally, the higher magnification revealed spiky projections in the sample with the highest cotrimaxazole concentration (Figure 6D). In the neomycin treatment of *C. portucalensis*, there were sparse cells (Figure 7A,B), with some cell elongation observed at the highest concentration (Figure 7B).

#### 3.2.7. Detection of Quorum Sensing

*C. portucalensis* also produces AHL type QS signals as assessed by the same methods for AHL detection as before (Figure 4). The *C. portucalensis* strain failed to activate the AHL-dependent response of violacein production in the narrow-range biosensor CV026, but activated the broad-range AHL-TraR-based reporter gene in biosensor NTL4 (pZLR4). The *C. portucalensis* strain showed a single elongated spot, which upon close inspection, appears as two overlapping joined spots. Upon close comparison with seven different AHL standards, clear co-migrations of either putative AHL spot from *C. portucalensis* with any AHL standard on the chromatogram was not discernable (Figure 4).

## 4. Discussion

*A. hydrophila* has been reported to resist different antibiotic classes such as quinolones, aminoglycosides, beta-lactams, tetracyclines, chloramphenicol, trimethoprim, and sulfonamides [22,95,96]. *A. hydrophila* strains expressing the AheABC efflux pump were able to export 13 substrates, including nine antibiotics, and the suppression of the efflux process by the inhibitor phenylalanine-arginine-β-naphthylamide, suggested the presence of additional systems leading to intrinsic resistance [97]. The antibiotics moxalactam and cefoxitin were demonstrated to inactivate the metallo-beta-lactamase CphA of an *A. hydrophila* strain by forming stable adducts via reaction with the active site residues [98]. Apart from harboring metallo-beta-lactamases and efflux pumps, naturally occurring *Aeromonas* spp. have been shown to be transformable and have a high incidence of horizontal gene transfer [99,100]. Shiga-like toxin production causing bloody diarrhea and hemolytic uremic syndrome is reported from *Aeromonas* spp. [101], and there is evidence that surface colonization upregulates the expression of the Shiga-like toxin genes [17].

The mortality rate for hospitalized patients with *Citrobacter* infections is reported to be 6.8% [29], and this figure rises to 18–56% for patients with *Citrobacter* bacteremia [30,102,103]. *C. freundii* strains are already known for extended β-lactamase and quinolone resistance [32,39,40,41,42,43]. *C. portucalensis* strains also are multidrug resistant [24,25], with a poultry isolate considered a “superbug” [24]. Making a rational choice of antimicrobial therapy for *Citrobacter* infections can be problematic, since they encode chromosomal, inducible *ampC* β-lactamase genes. High levels of constitutive expression due to mutational changes confer resistance to multiple antibiotics [102,104]. Additional plasmid-mediated determinants of resistance may co-exist [103]. Multidrug resistant *C. freundii* clinical isolates resist up to 12 antibiotics, including penicillins, cephalosporins, carbapenems, and fluoroquinolones [48]. Extreme drug resistance is also known [49]. Furthermore, the genome of the leafy vegetable strain of *C. portucalensis* possesses aminoglycoside, beta-lactam, chloramphenicol, sulfonamide, tetracycline, and trimethoprim resistance genes [25], whereas the poultry superbug *C. portucalensis* encodes 13 antibiotic resistance genes, enabling resistance to eight distinct groups of antibiotics [24]. The *mcr-3* gene encoding resistance to the last resort antibiotic colistin, is also disseminated universally in both Enterobacteriaceae (which include *Citrobacter* spp.) and *Aeromonas* spp., and the latter is a potential reservoir for this gene [105]. We have used pre-loaded discs and stock concentration tests to assess the resistance at different concentration ranges, primarily to establish upper limits (observe if any effect is seen even at concentrations much higher than allowed for clinical applications). Our results show *A. hydrophila* and *C. portucalensis* resist many more classes of antibiotics than predicted by bioinformatics analysis using the Resistance Gene Identifier (RGI) tool [94] (Table 3), suggesting that the database may be incomplete. The existing international criteria for multi-drug resistance (MDR) in the Enterobacteriaceae is the non-susceptibility to at least three classes of antibiotics [106]. Therefore, both the isolates in this study qualify as multidrug resistant. The overexpression of genes encoding for ATP-binding cassette (ABC) transporter membrane proteins or multidrug efflux pumps, which enable the export of drugs out of the cell is a prevalent cause of MDR [107,108,109]. Analysis of the genomes using the RGI server suggest that efflux pumps may be present in both isolates [17]. Future studies should address the mechanisms of MDR in the isolated *A. hydrophila* and *C. portucalensis* strains.

Biofilms are directly related to virulence [110]. Sessile cells are resistant to antibiotics and various disinfectants [111]. Frequently, the results of in vitro antimicrobial studies do not correlate with therapeutic results, since most studies assess resistance in the planktonic phase, and biofilms are not taken into account [12]. Biofilm phases of most bacteria are able to resist antibiotics at concentrations hundreds or thousands of times higher than the corresponding planktonic phases [44,46,65,66,67,68,69]. Cotrimoxazole was reported to be effective against *A. hydrophila* and *Citrobacter* spp. in the planktonic phase [112]. It has been reported to cure *C. freundii* infections even when other drugs have failed [113]. Over 70% of *Citrobacter* isolates in a 11-year single center study were reported to be susceptible to cotrimoxazole [60]. Neomycin has also been reported to clear infections caused by *C. freundii* [61]. Studies also report most food-associated strains and 50–65% of animal-associated strains of *A. hydrophila* as being susceptible to neomycin and cotrimoxazole [62,63]. Hence, the susceptibility of our strains to neomycin and cotrimoxazole in the planktonic phase was not surprising.

However, biofilms are expected to be more resistant. In fact, biofilms of *C. freundii* can be eradicated only by chlorite based disinfectants [114], which are toxic to humans. Therefore, surface colonization by RIT668 and RIT 669 poses a significant risk in case humans do get infected with them, in view of their multidrug resistance and biofilm formation, as well as toxin upregulation in biofilms formed on plastics [17]. Plastics, particularly microplastics, can act as vectors for bacterial pathogens [115] and aquatic animals ingest significant quantities of microplastics [116]. Thus, the environmental transmission of plastic waste from infected aquatic animals may expose humans to antibiotic resistant pathogens. Our strains were obtained from infected turtles, where they caused fatal infections. Additionally, at least one case where reptile and clinical strains of *Aeromonas* spp. were identical is known [18]. Therefore, the potential for zoonotic transmission of RIT668 and RIT669 must be considered.

SEM can be used to observe the overall shape of microorganisms composing the biofilm and their spatial organization, which can resolve biofilm growth on surfaces containing junctions between materials [117,118]. The optical sectioning of biofilms has provided the insight that biofilm structure depends on the type of bacterium [119]. *A. hydrophila* biofilms in this study did not show any specialized structures. However, the fibers connecting cells of *C. portucalensis* reported here are similar to those formed by enteroaggregative *Escherichia coli* in mixed diarrhea-associated biofilms with *C. freundii* and whose formation is considered to be mediated by *F. pili* [8]. Along with the morphological changes of the cells, the SEM analysis of samples in this study also revealed structurally modified cellular products surrounding the biofilm clusters, as reported by other authors [120].

The *luxI* gene codes for the AHL-synthase enzyme, while the LuxR is the AHL-receptor [121]. The bioinformatics analysis of each genome revealed that *A. hydrophila* RIT668 possesses a single *luxI* and three *luxR* homologs, whereas *C. freundii* RIT669 encodes for no identifiable *luxI* homologs but contains a single luxR homolog. However, in our experiments, both *A. hydrophila* and *C. portucalensis* produced a dose-dependent response in the AHL-dependent NTL4 (pZLR4) which uses the TraR receptor (Figure 4B). *A. hydrophila* and *A. salmonicida* strains have been shown to express homologs of the *luxI* and *luxR*, and produce C4 AHL as the major and C6 AHL as the minor quorum sensing signals [122]. Here we show that *A. hydrophila* RIT668 produces a single AHL, which co-migrates with *N*-(3-Hydroxyoctanoyl)-L-homoserine lactone. AHL-type QS signals are not produced by certain *C. freundii* strains [123]. Diketopiperazines (DKPs) are known to be produced in some *C. freundii* strains, which may activate the AHL-sensing proteins, but no biosensor response was observed in this study with biosensor CV026 (Figure 4A), which is known to cross-activate with DKPs [124]. In contrast, the broad-range TraR-dependent biosensor was activated in a dose-dependent manner (Figure 4B) and TLC analysis showed an elongated spot that upon close examination appear as two but overlapping AHL-like signal spots (Figure 4C). Biofilms of our isolates are not eradicated by even 1000 µg/mL of cotrimoxazole or neomycin. Owing to the well-known hematological toxicity of cotrimoxazole [125], as well as nephrotoxicity and ototoxicity of neomycin [126], such high dosages are precluded from clinical use. Biofilm formation is coordinated by QS mechanisms and since biofilms are harder to treat with antibiotics, the inhibition of QS could be a viable antibacterial strategy [127]. Chestnut honey and vanillin have been reported as having anti-biofilm and anti-QS effects on *A. hydrophila* [128,129]. N-acetylcysteine (NAC) linked to polymers offers a way to block biofilm formation by both *A. hydrophila* and *C. freundii* strains [6]. In view of the multidrug resistance of our isolates, anti-QS and anti-biofilm agents might offer effective alternatives to antibiotics.

## 5. Conclusions

*A. hydrophila* RIT668 and *C. portucalensis* RIT669 are multidrug resistant, and can be controlled only by neomycin and cotrimoxazole in the planktonic phase. However, the eradication of biofilms of either organism may need >> 1000 µg/mL of neomycin or cotrimoxazole, effectively meaning that neomycin or cotrimoxazole administration is not a clinical option, and indirect approaches to suppress biofilms may be necessary. *A. hydrophila* produces *N*-(3-Hydroxyoctanoyl)-L-homoserine lactone quorum sensing signals, but their significance for virulence or antibiotic resistance in RIT668 will require further investigations. *C. portucalensis* RIT669 activated our broad range AHL-dependent whole cell biosensor but further work is need to identify the potential two AHLs identified as well as the biosynthetic pathway used since a *luxI* homolog was not readily located in our whole genome sequence of this strain. The emergence of multi-drug resistance in *Aeromonas* spp. strains in water environments [130] and resistance determinants against last resort antibiotics, such as colistin [105,131] make *A. hydrophila* species increasingly dangerous pathogens. Multidrug resistant *C. freundii* which produce both carbapenemase and metallo-beta-lactamase have been reported, which resisted all tested antibiotics except tetracycline have been isolated in China [132], while a potentially zoonotic superbug *C. portucalensis* strain is known [24]. This suggests *C. portucalensis* and *C. freundii* strains are also emerging as a significant risk. The capacity for plastic colonization, multidrug resistance, and biofilm formation suggests that our turtle isolates, *A. hydrophila* RIT668 and *C. portucalensis* RIT669, are potential zoonotic pathogens, with heightened risks for patients living with polymer implants/IMDs. However, the mechanisms of multidrug resistance in these isolates is unknown and warrant further investigation.

## Figures and Tables

**Table 1:** Antibiotic susceptibility tests for *A. hydrophila* and *C. freundii* on Mueller-Hinton agar, using BD diffusion discs, pre-loaded with fixed amounts of antibiotics. Sulfamethoxazole * = sulfamethoxazole 23.75 μg + trimethoprim 1.25 μg. The inhibition zone diameters for sensitivity are based on the CLSI recommendations for Enterobacteriaceae [81]. ^#^ denotes EUCAST breakpoint for Enterobacterales. * denotes the values given by the following standard, https://microbeonline.com/novobiocin-susceptibility-test-principle-procedure-and-interpretations/. ^ denotes EUCAST breakpoints for *Staphylococcus* spp. The EUCAST numbers are taken from [81]. The responses are recorded as S = susceptible, R = resistant and I = intermediate.

Antibiotic	Amount of Antibiotic (μg)	Minimum Inhibition Zone Diameter (mm) for Sensitivity	*A. hydrophila*		*C. portucalensis*	
			Zone of Inhibition (mm)	Response	Zone of Inhibition (mm)	Response
Gentamicin	10	15	0	R	0	R
Tetracycline	30	15	0	R	0	R
Doxycycline	30	14	0	R	0	R
Kanamycin	30	18	0	R	0	R
Streptomycin	10	15	0	R	0	R
Tobramycin	10	15	0	R	0	R
Neomycin	30	12 ^#^	13	S	16	S
Novobiocin	30	12 *	5	R	0	R
Erythromycin	15	21 ^	10	R	7	R
Sulfamethoxazole + Trimethoprim	23.75 + 1.25	16	18	S	12	I
Penicillin	10 U	15	6	R	0	R
Sterile disc	0	N/A	0	N/A	0	N/A

**Table 2:** ‘Stock concentration’ susceptibility tests using blank BD diffusion discs spotted with stock concentrations of antibiotics for *A. hydrophila* and *C. portucalensis* grown on tryptic soy agar (TSA). The minimum zone of inhibition diameters are based on the values recommended by the CLSI for Enterobacteriaceae [81]. * denotes recommended for Rifampin by the CLSI for *Enterobacter* spp. ^#^ denotes EUCAST breakpoint for *Staphylococcus* spp. ^ denotes EUCAST breakpoint for *Streptococcus* groups. ^§^ denotes EUCAST breakpoint for Enterobacterales. Cotrimoxazole = trimethoprim with sulfamethoxazole (ratio 1:5). The EUCAST numbers are taken from [81]. The responses are recorded as S = susceptible, R = resistant and I = intermediate.

Antibiotic (Concentration in mg/mL)	Amount on Disc (μg)	Minimum Inhibition Zone Diameter for Sensitivity (mm)	*A. hydrophila*		*C. portucalensis*	
			Zone of Inhibition (mm)	Response	Zone of Inhibition (mm)	Response
Tetracycline (30)	600	15	31	S	30.7	S
Rifamycin (30)	600	20 *	0	R	0	R
Cefaclor (50)	1000	18	0	R	0	R
Fusidic acid (12.5)	500	24 ^#^	0	R	0	R
Kanamycin (50)	1000	18	0	R	0	R
Clindamycin (50)	1000	17 ^	8	R	0	R
Neomycin (30)	600	12 ^§^	22.5	S	23.5	S
Cotrimoxazole (30)	600	16	30.5	S	35	S
Control	N/A	N/A	0	N/A	0	N/A

**Table 3:** Antibiotics used in this study (Kirby–Bauer disc diffusion) for *A. hydrophila* and *C. portucalensis* their drug classes and therapeutic targets. R = resistant, and S = susceptible. Resistance Gene Identifier (RGI) [94] predicts resistance based on the genome information; Yes = resistance predicted; No = resistance not predicted. The predictions are drawn from published data [17].

Antibiotic	Antibiotic Class/Family	Therapeutic Target	*A. hydrophila*		*C. portucalensis*	
			Response	RGI Prediction	Response	RGI Prediction
Gentamycin	Aminoglycoside	30S ribosomal subunit (protein synthesis)	R	No	R	Yes
Tetracycline	Tetracycline	Aminoacyl tRNA binding to RNA-ribosome complex (30S subunit; protein synthesis)	R, but S at 600 μg	Yes	R, but S at 600 μg	Yes
Doxycycline	Tetracycline	Aminoacyl tRNA binding to RNA-ribosome complex (30S subunit; protein synthesis)	R	Yes	R	Yes
Kanamycin	Aminoglycoside	30S ribosomal subunit (protein synthesis)	R	No	R	Yes
Streptomycin	Aminoglycoside	30S ribosomal subunit (protein synthesis)	R	No	R	Yes
Tobramycin	Aminoglycoside	30S ribosomal subunit (protein synthesis)	R	No	R	Yes
Neomycin	Aminoglycoside	30S ribosomal subunit (protein synthesis)	S	No	S	Yes (R predicted)
Novobiocin	Aminocoumarin	DNA synthesis	R	No	R	Yes
Erythromycin	Macrolide	Growth	R	No	R	Yes
Penicillin	Beta-lactams	Final stages of cell wall synthesis	R	No	R	Yes
Rifamycin	Ansamycin	DNA-dependent RNA polymerase	R	No	R	Yes
Cefaclor	Cephalosporin	Peptidoglycan synthesis	R	Yes	R	Yes
Fusidic acid	Fusidane	Translocation of elongation factor G (Protein synthesis)	R	No	R	No
Clindamycin	Lincomycin	50S ribosomal subunit (protein synthesis)	R	No	R	No
Cotrimoxazole	Sulfonamides	Dihydropteroate synthase (DHPS)	R, but S at 600 μg	No	R, but S at 600 μg	No

**Table 4:** Representation of the overall results of the MIC, MBC, and MBEC assay to evaluate the efficacy of cotrimoxazole and neomycin sulfate on the two organisms, *A. hydrophila* and *C.* *portucalensis*. Inoculum = 10^6^ CFU/mL.

Assay	*A. hydrophila* RIT668		*C. portucalensis* RIT669	
	Cotrimoxazole (μg/mL)	Neomycin (μg/mL)	Cotrimoxazole (μg/mL)	Neomycin (μg/mL)
MIC	500–1000	32.3–62.5	7.8–15.6	7.8–31.3
MBEC	>1000	>1000	>1000	>1000

## Supplementary Materials

The following are available online at https://www.mdpi.com/2076-2607/8/11/1805/s1, Figure S1: FASTME Phylogenetic tree of 15 strains related to strain RIT668 based on GBDP distances, Figure S2: Fisher individual 95% confidence intervals for the MBEC values from the neutralizer plate of *A. hydrophila* RIT668, Figure S3: FASTME Phylogenetic tree of 13 strains related to strain RIT669 based on GBDP distances, Figure S4: Fisher individual 95% confidence intervals for the MBEC values from the neutralizer plate of *C. portucalensis* RIT669.
