# Supplementary material for: Aeromonas hydrophila RIT668 and Citrobacter portucalensis RIT669—Potential Zoonotic Pathogens Isolated from Spotted Turtles
Source: Microorganisms. 2020 Nov 17;8(11):1805. doi: 10.3390/microorganisms8111805 (PMC7698337; doi:10.3390/microorganisms8111805)
Supplement: Supplementary file 1 [file microorganisms-08-01805-s001.pdf]

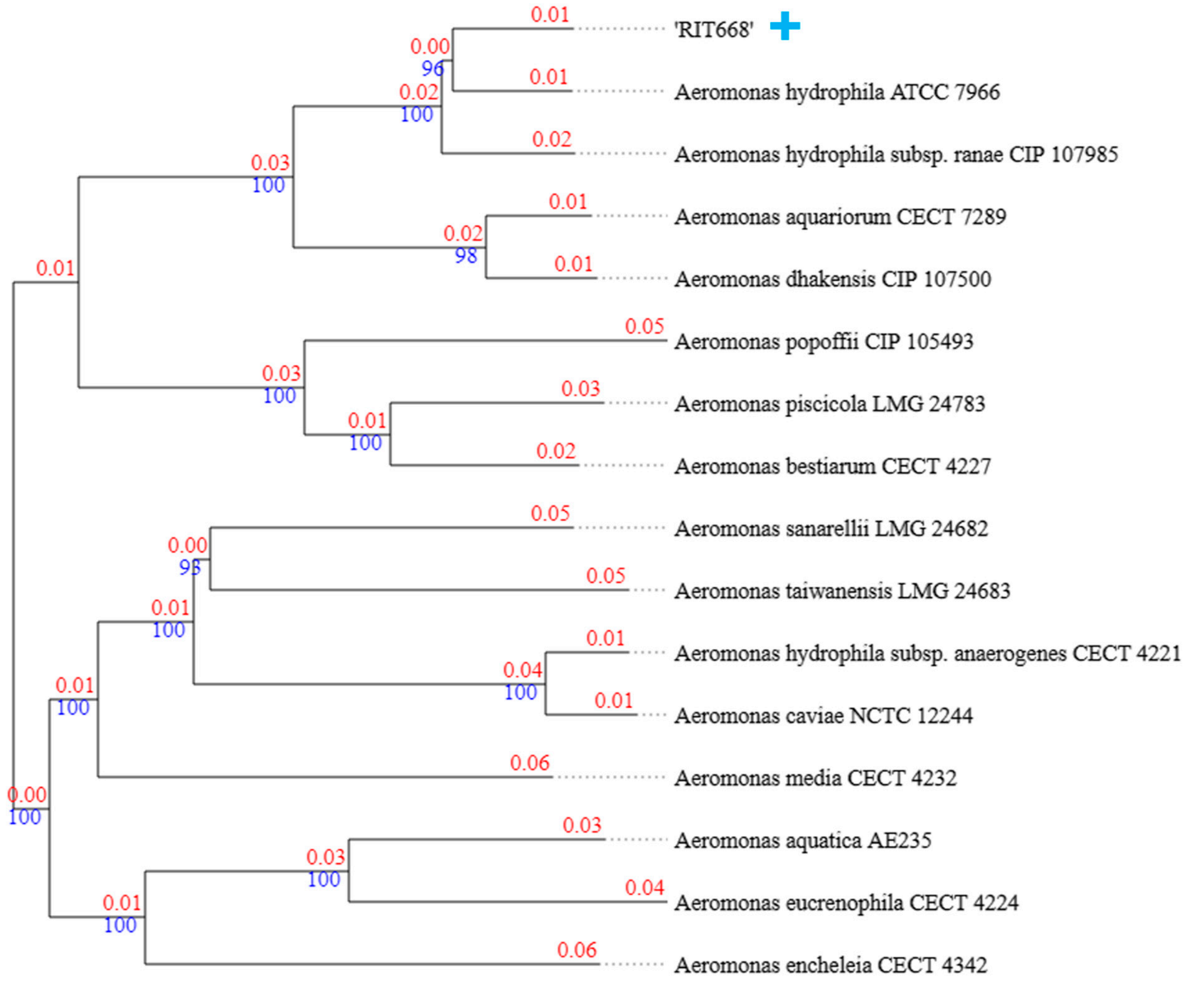

Figure S1. FASTME Phylogenetic tree of 15 strains related to strain RIT668 based on GBDP distances. Strain RIT668 is marked with a blue plus sign. Bootstrap values are in blue, and branch lengths are in red. Average branch support for this tree = 99.0%.

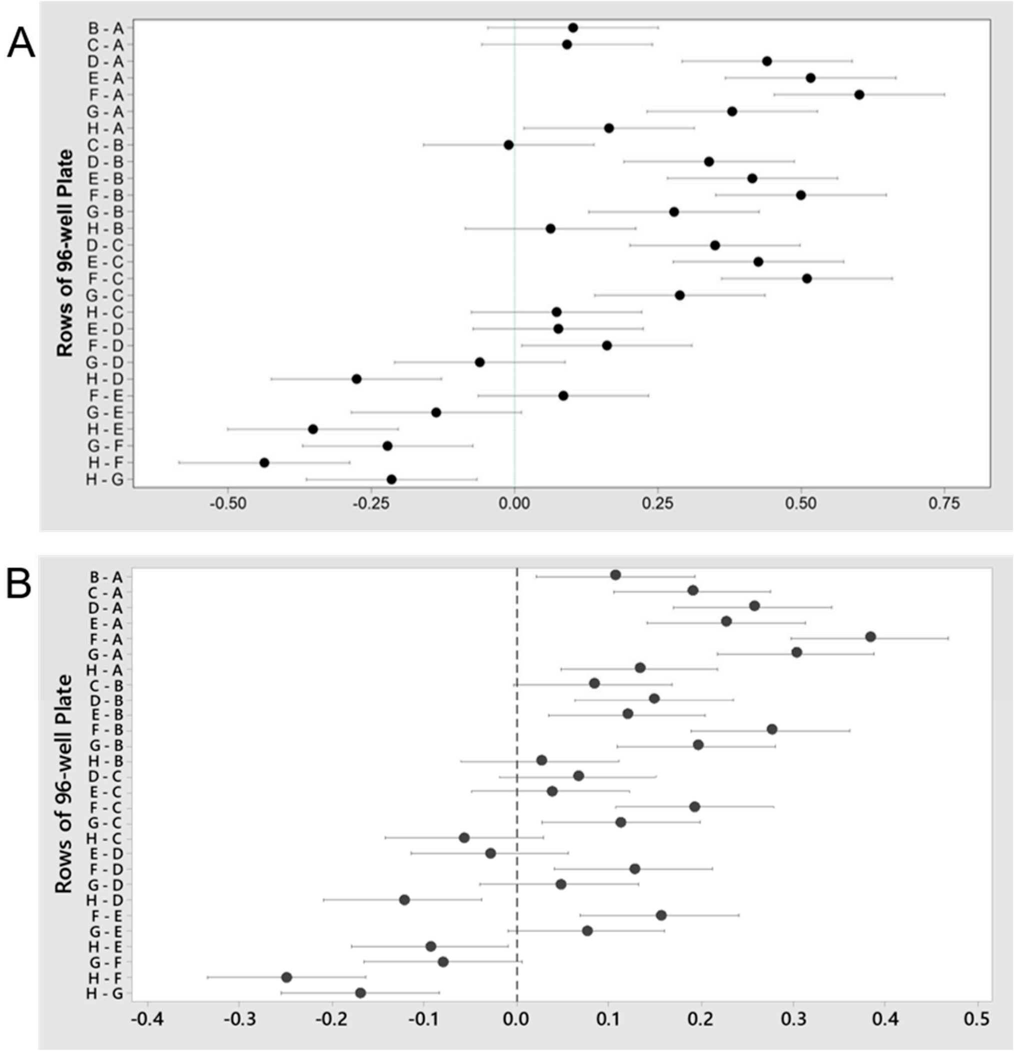

Figure S2. Fisher individual 95% confidence intervals for the MBEC values from the neutralizer plate of *A. hydrophila* containing **(A)** cotrimoxazole; the exact value of the MBEC was indeterminate as no tested concentration completely eradicated the biofilms. However, a significant difference between 250 and 125 μg/mL (rows C and D) indicate an area of interest, and **(B)** neomycin; the exact value of the MBEC was indeterminate as no tested concentration completely eradicated the biofilm. However, a moderate trend relating to higher concentrations being more effective is visible with the concentrations evaluated.

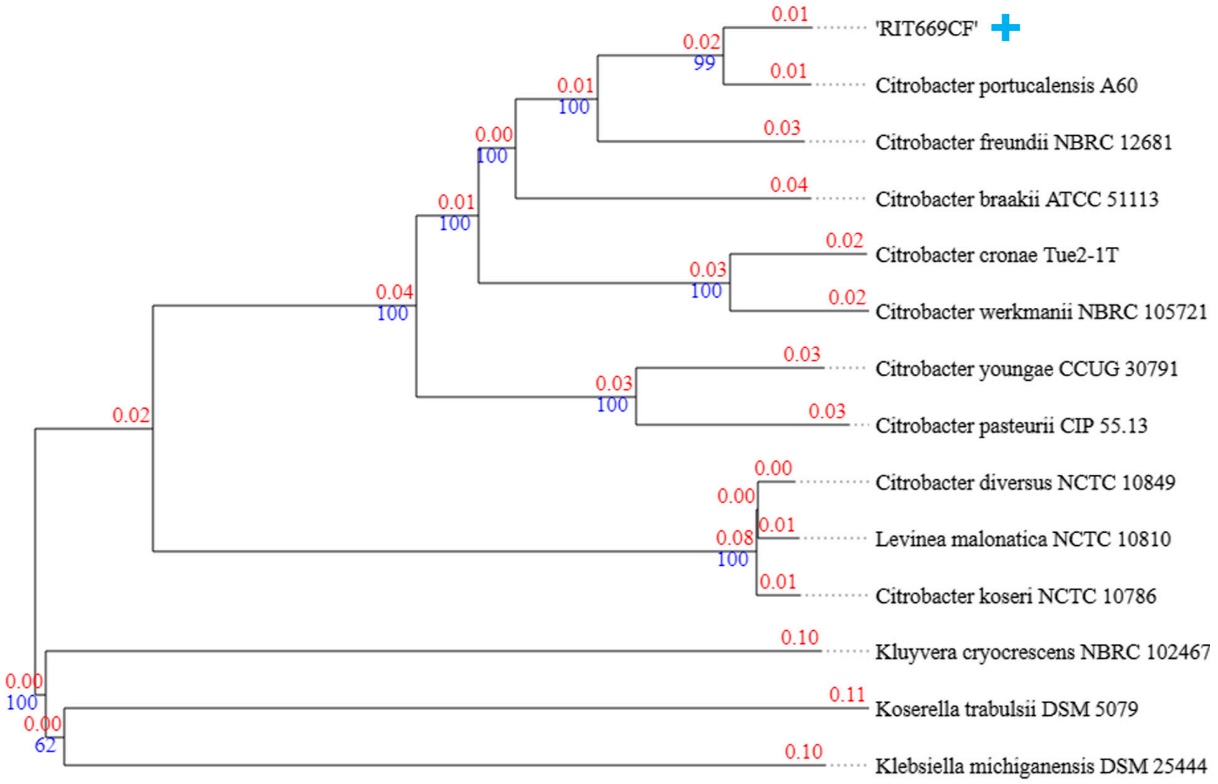

Figure S3. FASTME Phylogenetic tree of 13 strains related to strain RIT669 based on GBDP distances. Strain RIT669 is marked with a blue plus sign. Bootstrap values are in blue, and branch lengths are in red. Average branch support for this tree = 91.7%.

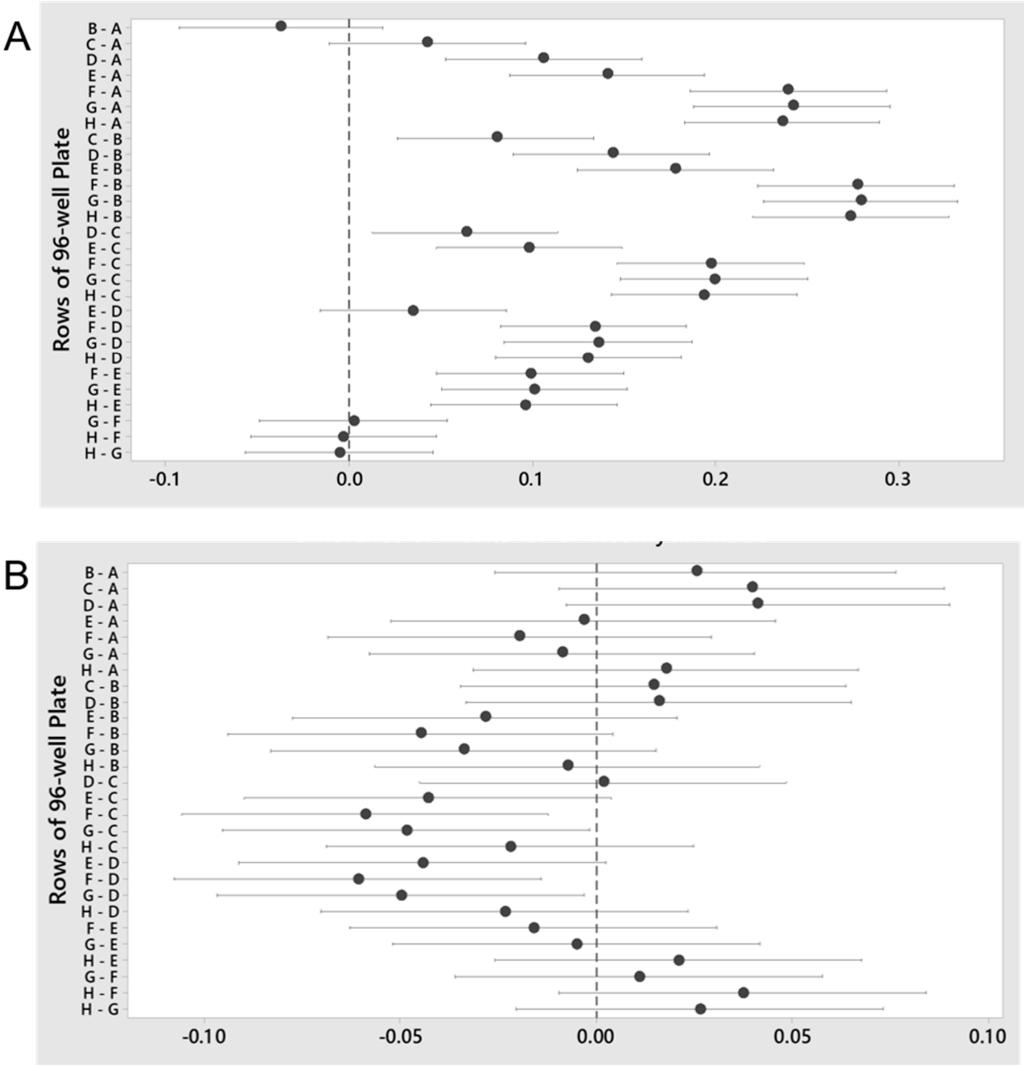

Figure S4. Fisher individual 95% confidence intervals for the MBEC values from the neutralizer plate of *C. portucalensis* containing **(A)** cotrimoxazole; the MBEC was indeterminate as no tested concentration completely eradicated biofilm growth. However, a moderate trend is visible with the concentrations evaluated, and **(B)** neomycin; the MBEC was indeterminate as no tested concentration completely eradicated biofilm growth.
